# Prevention and treatment of osteoporosis in women

**DOI:** 10.1177/20533691221139902

**Published:** 2022-11-10

**Authors:** John Stevenson, in collaboration with the medical advisory council of the British Menopause Society

**Affiliations:** National Heart and Lung Institute, 90897 Imperial College London, Royal Brompton Hospital, London, UK

## Summary

This guidance regarding estrogen and non–estrogen-based treatments for osteoporosis responds to the controversies about the benefits and risks of individual agents. Treatment choice should be based on up to date evidence-based information and targeted to individual women’s needs.

## Introduction

Osteoporosis is very much a disease of older women affecting 1 in 3 women compared to 1 in 5 men. Osteoporosis is as a skeletal disorder characterized by compromised bone strength predisposing to an increased risk of fracture. Fractures of the wrist, hip and vertebrae, which are the main clinical manifestations of osteoporosis, have enormous impact on quality of life, result in significant economic burden and are associated with considerable excess mortality. The annual number of hip fractures in the UK due to osteoporosis has risen by 44% from 70,000 in 2006 to >100,000 in 2020.^1^ Pharmacological and non-pharmacological therapies will be examined (Table 1).

**Table 1.** Interventions for the prevention and treatment of osteoporosis.

• Hormone replacement therapy
Estrogen alone
Estrogen plus progestogen
Tibolone
• Bisphosphonates
Alendronate
Risedronate
Ibandronate
Zoledronate
• Denosumab
• Raloxifene
• Parathyroid hormone peptides
• Romozosumab
• Calcium and vitamin D
• Exercise

## Pharmacological interventions

All pharmacological interventions except for parathyroid hormone act mainly by inhibiting bone resorption. Very few data exist about long-term efficacy for reducing fractures (that is, more than 10 years of treatment) and about the safety of combinations of therapy. In many of the controlled studies, the placebo group received calcium and vitamin D supplements.

### Hormone replacement therapy

There is evidence from randomized controlled trials including the Women’s Health Initiative (WHI) that hormone replacement therapy (HRT) reduces the risk of both spine and hip as well as other osteoporotic fractures even in women at low risk^2^ as well as in those with established osteoporosis.^3^ The ‘standard’ bone conserving doses of estrogen were considered to be oral estradiol 2 mg, conjugated equine estrogens 0.625 mg and transdermal 50 mcg patch. However, it is now evident that lower doses also conserve bone mass.^4^ Epidemiological studies have suggested that for HRT to be an effective method of preventing fracture, continuous use is required. However, it has been shown that just a few years treatment with HRT around the time of menopause may have a long term effect on fracture reduction.^5^ European regulatory authorities (December 2003) advised that HRT should not be used as a first line treatment for osteoporosis prevention as the risks outweigh the benefits.^6^ This view was robustly challenged,^7^ but despite a subsequent wealth of further evidence, the regulatory authorities have not revised their position. Yet HRT is an effective, safe and inexpensive treatment. Whilst alternatives are available for the treatment of osteoporosis in elderly women, estrogen still remains the best and safest option for prevention, particularly in younger (aged less than 60 years) and/or symptomatic women.^8^ The initiation of HRT for fracture prevention in women over 60 needs the starting dose to be tailored to the age of the woman.^9^

Estrogen-based therapy remains the treatment of choice in women with premature ovarian insufficiency.^10^ No clinical trial evidence attests the efficacy or safety of the use of non–estrogen-based treatments, such as bisphosphonates, denosumab or raloxifene, in these women.

Although some women will be happy to take HRT for life to manage osteoporosis, others may view treatment as a continuum of options for bone protection and may wish to change to other agents such as a bisphosphonate because of the possible small increase in risk of diagnosis of breast cancer associated with the long-term use of combined HRT.

### Bisphosphonates

Bisphosphonates are chemical analogues of naturally occurring pyrophosphates thus allowing them to be integrated into bone where they have a direct effect on osteoclasts, thereby reducing bone resorption. This makes metabolism an extremely slow process, indeed the skeletal half-life of alendronate has been estimated as high as over 12 years. There are concerns about effects on the fetal skeleton and bisphosphonates are not advised in women with fertility aspirations. However, they are widely used for postmenopausal women and are effective in fracture prevention.^11^

Alendronate, risedronate, ibandronate and zoledronate are all used in the treatment of postmenopausal osteoporosis, and are also used in corticosteroid-induced osteoporosis.

The question of how long to prescribe a bisphosphonate has not been fully clarified yet. There are concerns about fatigue damage due to oversuppression of bone remodelling with long-term use in some individuals leading to femoral fragility fractures and also development of osteonecrosis in the jaw. Although such adverse effects are very rare (around 1 in 5000 women per year), 5 years of treatment with a 1-to 2-year ‘holiday’ have been proposed to try to reduce these risks.^12^ This may not be applicable in glucocorticoid-induced osteoporosis because of the very long skeletal retention time of bisphosphonates, any very long term adverse effects still remain unknown. They should therefore be avoided where possible in younger (e.g. aged <65 years) patients.

The vast majority of reports of osteonecrosis of the jaw refer mainly to intravenous bisphosphonates used in the oncological setting. Very few cases have been reported in women using oral bisphosphonates for osteoporosis. These cases usually, but not exclusively, follow dental extractions, and dental review could be considered in women with significant dental disease before initiation of bisphosphonates therapy.

Fragility fractures of the femoral shaft have now been increasingly reported in patients on long term bisphosphates. These occur spontaneously but may be preceded by thigh pain and the presence of cortical ‘beaking’ on plain radiographs of the femur.^13^

#### Alendronate

Alendronate reduces vertebral and non-vertebral fractures by 50% in randomized controlled trials in osteoporotic women.^14^ The dose for osteoporosis treatment is 70 mg once weekly.

#### Risedronate

Risedronate reduces vertebral and non-vertebral fractures in randomized controlled trials.^15^ The dose for treatment of established disease is 35 mg once weekly.

#### Ibandronate

Ibandronate has been shown to reduce the incidence of vertebral, but not non-vertebral fractures by 50% in randomized controlled trials undertaken in postmenopausal women.^16^ The dose is 150 mg orally once a month, or 3 mg by intravenous injection every 3 months.

#### Zoledronate

Zoledronate has been shown to reduce both spine and hip fracture osteoporotic incidence in the elderly.^17^ It is given in a dose of 5 mg as an annual intravenous infusion. It is the most potent of the currently used bisphosphonates and hence has the highest rate of adverse effects mainly after the first infusion, which can also include atrial fibrillation and inflammatory eye disease. Creatinine clearance should be confirmed to be >35 mL/min prior.

### Denosumab

Denosumab is a monoclonal antibody to receptor activator of nuclear factor k-B ligand (RANK-L), a major signal promoting osteoclast activity. It is as effective as the bisphosphonates in terms of spine and hip fracture reduction in osteoporotic women,^18^ but also has similar adverse effects in terms of osteonecrosis of the jaw and femoral fragility fractures. However, it is not retained in the skeleton and may be a safer option for younger women. Because RANK-L also has a role in the immune system, denosumab is associated with an increased risk of infections and should be avoided in patients with increased susceptibility. There is evidence of an accelerated loss of bone on discontinuation of denosumab and hence a concern about an increased risk of fractures.^19^ It may be prudent to introduce another treatment when discontinuing denosumab.

### Selective estradiol receptor modulators

These compounds possess estrogenic actions in certain tissues and anti-estrogenic actions in others. Raloxifene is licensed for the prevention of osteoporosis-related vertebral fracture. It reduces vertebral but not non-vertebral fracture by around 35%.^20^ The dose is 60 mg/day. It also reduces the risk of breast cancer to the same extent as tamoxifen.^21^ Side effects include hot flushes and calf cramps. It was thought that it could be cardioprotective from its effects on lipids, and the Raloxifene Use for the Heart (RUTH) study found that it did reduce the risk of coronary heart disease in those initiating treatment below age 60 years, but it increased the risk of fatal stroke and venous thromboembolism.^20^

### Parathyroid hormone peptides

Recombinant 1-34 parathyroid hormone (teriparatide), given as a subcutaneous daily injection of 20 μg, reduces vertebral and non-vertebral fractures in postmenopausal women with osteoporosis.^22^ It has been shown to reduce the risk of vertebral and non-vertebral, but not hip, fractures. Because it costs considerably more than other options, it is reserved for patients with severe osteoporosis who are unable to tolerate, or seem to be unresponsive to, other treatments.

### Romozosumab

Romozosumab is a monoclonal antibody which binds sclerostin, a natural inhibitor of the Wnt/LRP pathway which is a major signal to osteoblasts to promote bone formation. Thus, blocking sclerostin action leads to an increase in bone formation.^23^ Romozosumab is given by subcutaneous injection every 2 weeks for a 12-month course.

## Non-pharmacological interventions

Advice should be given to menopausal women regarding lifestyle modification and bone health. This should include information on a balanced diet, adequate calcium and vitamin D intake, exercise and smoking cessation as well as avoidance of excessive alcohol intake.

### Calcium and vitamin D

Provision of adequate dietary or supplemental calcium and vitamin D can be a part of osteoporosis management. The effects of calcium and vitamin D supplements alone or in combination on fracture, however, are contradictory and may depend on the study population.^24,25^ Hip fracture reduction has been shown in elderly women in residential care, but such women may be more frail, have lower dietary intakes of calcium and vitamin D and are at higher risk of fracture than those living in the community in whom fracture reduction has not been shown.

Furthermore, the Women’s Health Initiative Study showed an increase in kidney stones in low-risk women taking calcium and vitamin D supplements,^26^ and there are controversial claims about increased cardiovascular risk being associated with such supplements. The British Menopause Society/Women’s Health Concern recommends a daily intake of 1000 mg calcium and 1000iu vitamin D.^27^

### Exercise

Although certain exercise regimens may increase bone density, a role for exercise in preventing osteoporotic fractures has not been convincingly shown.^28^ Exercise regimens can be helpful in the management of established osteoporosis. The benefits are mainly related to increased wellbeing, muscle strength, postural stability and a reduction of chronic pain rather than an increase of skeletal mass. Exercise has to be structured carefully because of concerns about falls and fractures.

## Summary practice points

1. HRT reduces the risk of both spine and hip as well as other osteoporotic fractures.
2. Estrogen remains the treatment of choice for osteoporosis prevention in experiencing menopause. It is especially important in those with premature ovarian insufficiency. It should be considered for osteoporosis prevention in women over the age of 60 who continue to benefit from HRT in terms of menopause symptom relief.
3. Bisphosphonates are effective for treatment of established osteoporosis, reducing both spine and hip fractures.
4. Bisphosphonates have a very long skeletal retention time and hence should be used with caution in younger postmenopausal women (e.g. those aged below 65 years).
5. Denosumab is an effective treatment for reducing spine and hip fractures in osteoporotic women.
6. Denosumab should be avoided in women with increased susceptibility to infections.
7. There may be an increased risk of fractures after denosumab discontinuation.
8. Provision of adequate dietary or supplemental calcium and vitamin D is a part of osteoporosis management.
9. The effects of calcium and vitamin D supplements alone on fracture reduction, however, are contradictory and may depend on the study population.
